# Diurnal behavioral outcomes and BBB integrity in adult male SHR and WKY rats

**DOI:** 10.1038/s41598-025-25585-1

**Published:** 2025-11-24

**Authors:** Pallavi Shrivastava, Oluchukwu D. Biose, Riya Dahal, Abdallah Jwayyed, Rukayat A. Raji, Huijing Xia, Eric Lazartigues, Ifechukwude J. Biose

**Affiliations:** 1 Cardiovascular Center of Excellence, Department of Pharmacology and Experimental Therapeutics, Louisiana State University Health Sciences Center, New Orleans, LA USA; 2 Tulane Center for Aging, Tulane University School of Medicine, New Orleans, LA USA; 3 School of Graduate Studies, Louisiana State University Health Sciences Center, New Orleans, LA USA

**Keywords:** Diurnal, Cognition, Neurobehavior, BBB, Hypertension, Diseases, Neuroscience

## Abstract

Day and night neurobehavioral tests were performed for Spontaneously Hypertensive rats (SHR) and Wistar Kyoto rats (WKY) to establish an optimal testing window. Also, blood–brain barrier (BBB) integrity in brain areas related to cognition was measured to determine strain-specific differences. Six months old male SHR and WKY were randomly divided into day (i.e., 9–11 a.m.) and nighttime (i.e., 7–9 p.m.) testing regimen. Neurobehavioral tests included: Y-maze spontaneous alternation, open field, novel object recognition (NOR), and tail suspension test (TST). BBB integrity was measured in the cortex, hippocampus and striatum. WKY showed higher recognition memory at night compared to SHR and daytime test groups. Working memory and exploratory behavior were higher for SHR at both testing times when compared to WKY. During the day and when compared to WKY, SHR showed significant depression-like behavior which was absent during the night. SHR exhibited a higher anxiety-like behavior at both testing times when compared to WKY. Immunofluorescent signal for FITC-Dextran significantly increased in brain regions of SHR compared to WKY, but BBB tight junction proteins decreased suggesting impaired BBB integrity in SHR. In sum, NOR and TST are best performed during the dark phase to observe actual baseline outcomes for SHR and WKY. Neurobehavioral outcome differences between strains are independent of BBB integrity.

## Introduction

Cognitive and mood-associated tests in animal models are the chief functional measures commonly used to deepen our understanding of the pathobiology of various neurodegenerative, developmental and psychiatric conditions as well as to test the efficacy of potential therapeutic strategies. Among widely used rat models for studying hypertension and its related comorbidities, spontaneously hypertensive rat (SHR) has garnered significant attention due to its genetic predisposition to elevated blood pressure and modelling of essential hypertension, making it an important rat strain for investigating cardiovascular disease and associated cognitive and mood-related behaviors^1–4^. Also, juvenile SHR is a valid model for attention deficit disorder (ADHD) as these rats exhibit neurobiological similarities to children with ADHD; these include inattention, hyperactivity, and impulsive behavior^5–8^. In contrast, the Wistar-Kyoto (WKY) rat serves as normotensive control for SHR since originally derived from selective inbreeding of the normotensive WKY rats^9,10^. Thus, WKY provides an appropriate basis for comparison in many experimental settings due to their genetic similarity with SHR.

However, three recent reports suggest that male WKY are less active during day-time cognitive and mood-associated tests with a potential for confounding test results when compared to male SHR^11–13^. Also, reports suggest that male Wistar rats are a better normotensive control for male SHR, than male WKY, as they show that Wistar rats were more cognitively superior to SHR and/ or WKY due to Wistar rat’s elevated spontaneous movement within test apparati^13–15^. Contrary to the notion that Wistar rats have a higher spontaneous activity than WKY, a recent report^13^ of a 24-h spontaneous activity show that male WKY and Wistar rats are comparable except at 4 PM (when WKYs have a higher spontaneous activity) and at 5–6 a.m. (when WKYs have significantly lower spontaneous activity). Equally, the spontaneous activity of SHR, WKY and Wistar rats are similar except at 12–1 p.m. and 12–1 a.m. when SHR shows significantly higher activity. Since WKY and SHR exhibit comparable spontaneous activity^13^ at 9–11 a.m. (i.e., light phase period typical for rodent behavior test performance in the laboratory) and at 7–10 p.m. (i.e., dark phase period convenient for a willing ‘night-shift’ experimenter), we hypothesize that there is no difference between light and dark phase test outcomes of cognition and mood-associated behaviors in commonly used neurobehavioral tests for male SHR and WKY.

Except for a few recent reports^16–18^, the majority of rodent tests are conducted during the light phase when rats are mostly inactive presumably due to increased sleep pressure. Hence, little attention has been paid to the effects of the time of day on the behavior of these rats, despite evidence that circadian rhythms can significantly influence both cognitive and emotional processes in rodents^18–24^. Nevertheless, to date there is no report on light and dark phase comparison for cognitive and mood-related behavioral tests results between SHR and WKY rats.

SHR develop spontaneous variable hypertension from 4 weeks of age which becomes stable from 12 weeks of age^25^. Rodents are considered in the adult phase of their life cycle at 16–24 weeks old and for SHR this coincides with a chronic period of stable hypertension^26,27^. Thus, behaviorally testing SHR at six months old captures a period after physiological adjustment to hypertension and any resultant changes in cognition, anxiety and depression-like behaviors can be gleaned. By testing distinct cohorts of rats during both the light and dark phases, we aim to elucidate whether time-of-day influences cognitive and/or behavioral outcomes in a way that could optimize experimental protocols and potentially facilitate reproducible results for cognitive and mood-associated outcomes in future studies. Understanding these temporal differences will help establish the most reliable window for conducting cognitive and mood-associated behavior tests in SHR and WKY rats.

## Material and methods

### Animal care and housing

A total of 26 male WKY and 27 male SHR, six months old, (Charles River Laboratories, Wilmington, MA), were used for this study. Animals were housed in groups of two per cage in a 20–26 °C temperature- and 30–70% humidity-controlled room. Animals were conditioned to 12-h light/ dark cycle with lights on at 6 a.m. and with unlimited access to food and water. Prior to experimentation, animals were housed for a minimum of one month to acclimate them to the vivarium conditions. The experiments described herein are reported in accordance with ARRIVE guidelines (https://arriveguidelines.org/) and approved by the Institutional Animal Care & Use Committee (IACUC; protocol #5211) of the Louisiana State University Health Sciences Center -New Orleans and in accordance with the National Institutes of Health guidelines. Distinct cohorts of animals were randomly assigned to two groups of test times (i.e., light phase or dark phase) per strain, as previously established^18^ with minor modification.

### Behavioral tests

**Fig. 1:**
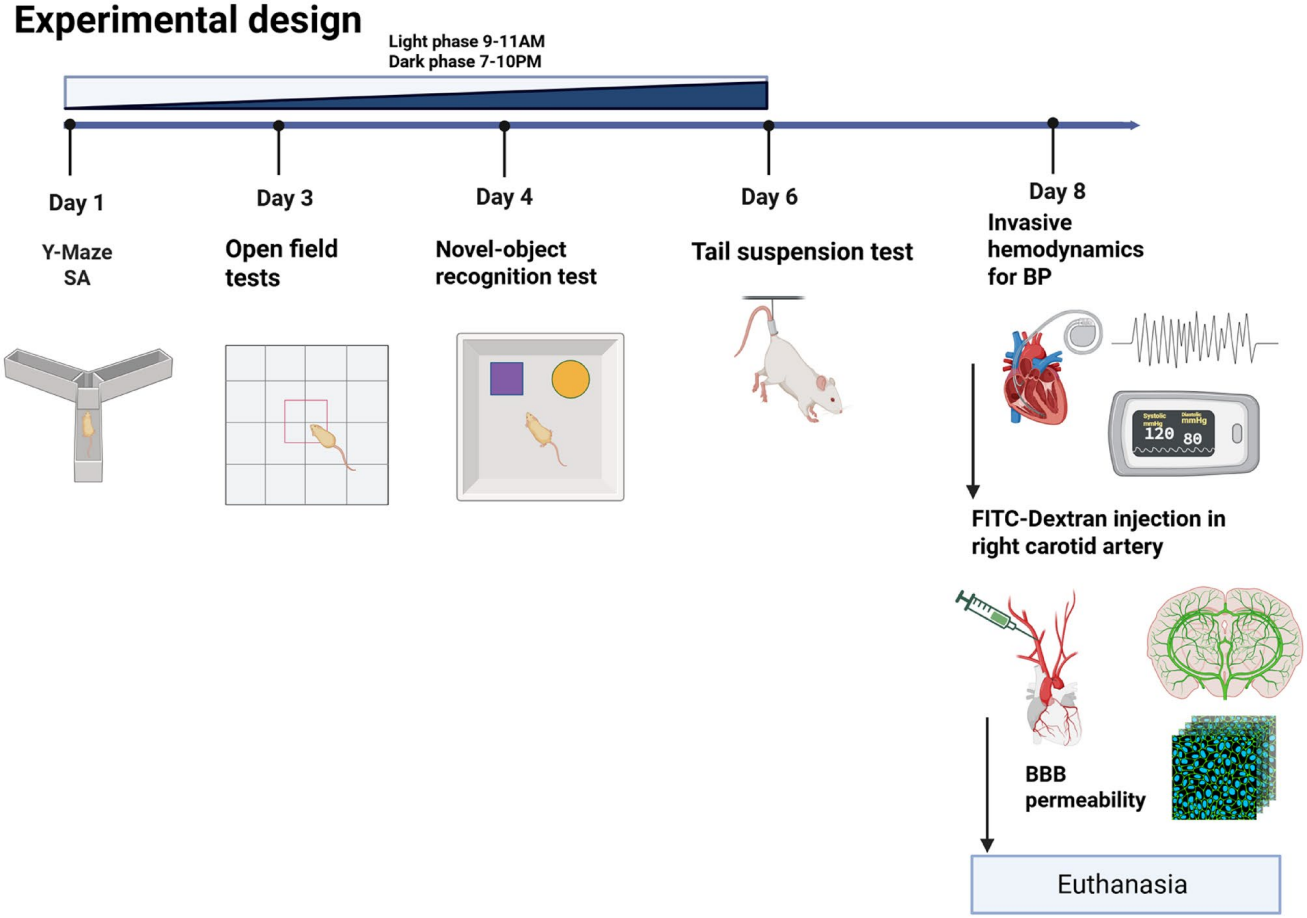
Schematic of experimental design. Timeline of diurnal behavioral testing in SHR and WKY rats. Rats were tested from 9–11 a.m. (light phase) and 7–10 p.m. (dark phase) for Y-maze, OFT, NOR and TST, followed by day-time in vivo hemodynamics measurement to confirm blood pressure phenotype and FITC-Dextran injection in right carotid artery. After 20 min of FITC-dextran injection animals were sacrificed, and brain harvested for permeability assay, RT-qPCR and immunohistochemistry of blood–brain barrier tight junction proteins.

All behavioral tests were performed by the same experimenter at 9–11 a.m. (light phase) and 7–10 p.m. (dark phase). The neurobehavioral laboratory dedicated to rat tests is in the same housing facility. Prior to each test, animals were allowed to acclimate to the room with dim visible light at 22 Lux (Cheffort Digital Lux Meter, AS803) for 45–60 min. Each apparatus was deodorized before and immediately after each test, urine and fecal boli were removed and the apparatus was cleaned with 70% isopropyl alcohol (MaxTite, USA). Live video recordings of animals were captured and analyzed with a CCD and ANY-maze Software (Stoelting, USA). A battery of neurobehavioral tests was conducted in the following order to reduce the impact of stress: Y-maze spontaneous alternation test, open field test, novel object recognition test, and tail suspension test as described briefly below (and summarized in the experimental timeline of Fig. 1).

*Y-maze spontaneous alternation test (Y-maze):* This test evaluates working/spatial memory by measuring correct spontaneous alternation^28^ between arms of the custom-made Y-maze apparatus (i.e., 12 cm wide, 50 cm long, and 40 cm tall; made from acrylic sheet, Piedmont Plastics, USA). Animals are placed into 1 of the 3 arms and allowed to explore the maze for 8 min. Data for total number of arm entries and percentage spontaneous alternation collated and analyzed as previously described^28^.

*Open field test (OFT):* The open field apparatus (Stoelting, USA) is used to assess the innate responses of the animal to open spaces whilst collecting data for locomotor activity and exploratory behavior (i.e., total distance moved) as well as anxiety-like behavior (i.e., cumulative time in the central zone)^29^. Animals were individually placed in the open field apparatus and allowed to explore the apparatus for a total of 10 min.

*Novel object recognition test (NOR):* Recognition memory was assessed in the NOR test. The recognition memory test is based on the natural tendency of rodents to investigate a novel object instead of a familiar one and their innate tendency to re-start exploring when presented with a novel environment. The OFT test performed a day earlier served as habituation phase for the NOR. For the familiarization phase, animals were individually placed in an open field apparatus for 10 min with two safe and non-toxic objects similar in color, texture and dimensions. For the discrimination phase, animals were returned to a holding cage and 30 min later the animal was returned to the open field apparatus after replacing one of the objects with a novel object which is different in color, texture and dimension. Discrimination index was calculated using the total time the rat spent exploring the familiar and novel objects in the discrimination phase as previously described^30^.

*Tail suspension tests (TST):* This measures despair or depression-like behavior in rodents. As previously established^28^, the animal was suspended by its tail for six minutes using a duct tape (Shurtape Technologies, LLC, USA) in the custom-made apparatus (i.e.,60 cm wide, 60 cm long, 75 cm tall made from acrylic sheet, Piedmont Plastics, USA). The distance of the animal was approximately 5 cm away from the floor of the apparatus and at least 20 cm from the bars on each side). Immobility time and number of immobile episodes were recorded.

### Blood pressure measurement

Systolic and diastolic arterial pressure were measured using an invasive hemodynamics technique^31^. Briefly, during the light phase and under 2% isoflurane anesthesia, a 2.0 -F transonic solid-state pressure transducer catheter (Millar^®^, USA) was surgically advanced to the root of the aorta through the right common carotid artery. Records of aortic systemic blood pressure were taken for a minimum of 30 s. Data were acquired and analyzed using LabChart (AD Instruments™, USA). Mean arterial blood pressure (MAP) was calculated from systolic and diastolic blood pressure (i.e., SP and DP, respectively) data using previously established formular MAP = DP + 1/3(SP – DP)^32^.

### In vivo FITC-Dextran permeability assay

FITC-Dextran (40 kDa; 45 mg/300 µl; Cat-53379, Sigma-Aldrich) was injected into the right carotid artery of each rat immediately after in vivo hemodynamics blood pressure measurement. Twenty minutes after FITC-Dextran injection, rats were sacrificed by decapitation under 3% isoflurane anesthesia and the whole brain harvested. Brain regions such as cortex, hippocampus and striatum were dissected from the right hemisphere and snap frozen. The left hemisphere was post-fixed in 4% paraformaldehyde (PFA) for histology. Homogenates of stated brain regions were prepared using cell lysis buffer with protease inhibitor (Cat-895347, R&D systems). Briefly, brain samples were weighed and lysed using Qiagen TissueLyser II with metal beads in 1 mL of lysis buffer using round bottom Eppendorf tubes. To collect 100 µl of supernatant, 2 mL of lysis buffer was added to homogenate and subsequently centrifuged at 13,500 rpm and 4 °C for 20 min. Supernatant was used for the quantification of FITC-dextran. Serial dilution of rat plasma samples with PBS was used to generate standard curve: Neat, 1:10, 1;100, 1:1000, 1:10,000, 1:100,000. The plate was read at excitation/emission: 485/535 nm on Varioskan LUX Multimode Microplate Reader. The FITC-Dextran permeability was quantified using permeability index (PI)^33^. The raw fluorescence unit (RFU) values from the plate reader was used to calculate PI after subtracting the blank values and according to the following^33^:

$${\text{Permeability Index}}\left( {{\text{mL}}/{\text{g}}} \right) = \, \left( {{\text{Tissue RFUs}}/{\text{g tissue weight}}} \right)/\left( {{\text{Plasma RFUs}}/{\text{mL Plasma}}} \right).$$

### Immunofluorescent staining for BBB tight junction proteins and visualization of FITC-Dextran

The left hemisphere, post-fixed in 4% PFA for 48 h, was dehydrated after 48 h in 30% sucrose. Hemispheres were individually embedded in optimum cutting temperature (OCT) cryostat embedding compound (Tissue-Tek, Torrance, CA) on dry ice to make tissue blocks for cryosection at − 20 °C using a cryostat (HM550, Thermo Fisher Scientific, Kalamazoo, MI, USA). Cryosections (20 μm) of the cortex, striatum and hippocampus were mounted on Fisherbrand™ Superfrost™ Plus Microscope Slides and stored at − 20 °C until used. Sections were processed for immuno-fluorescent histology. Briefly, sections were marked using hydrophobic barrier pap pen (Catalog number R3777, Thermo Scientific) and washed in phosphate-buffered saline (PBS), and then incubated in blocking buffer (1% BSA + 0.5% Trition-X + 0.02% Tween-20 in 1X PBS) at room temperature for 90 min. Sections were differently incubated overnight at 4 °C with primary antibodies: Claudin-5 polyclonal antibody (1:500, Rabbit, PA5-99415, Invitrogen) and Occludin monoclonal antibody (1:250, Mouse, 331594, Invitrogen). Following primary antibody incubation, sections were washed 3 times with 1X PBS for 10 min each and then incubated in Alexa Fluor-594 secondary antibodies (A11012 and A-11005, Invitrogen) with host compatibility. Sections were incubated in DAPI solution (1:10,000) for 10 min and mounted with Aqua-Mount mounting media (Epredia, cat-13800) and held at 4 °C until microscopy. Micrographs were acquired using ECHO-Revolve fluorescent microscope with 40× objective lenses. Green channel (i.e., FITC) was used to visualize FITC-Dextran and red channel (i.e., TRITC) for Claudin-5 and Occludin stains. Blue Channel (i.e., DAPI) was used to visualize cell nuclei. Micrographs of six sections per rat were quantified for % area of region of interests (ROI) using ImageJ software (National Institutes of Health, Bethesda, MD). The extravasation of the FITC–dextran was identified as specific green fluorescence occurring outside the vessel.

### Reverse transcription quantitative polymerase chain reaction (RT-qPCR) for BBB tight junction genes expression

Gene expression levels of BBB tight junction proteins (i.e., Claudin-5 and Occludin) in cortex, hippocampus and striatum were measured by RT-qPCR (SYBR green assay). RNA was isolated using RNeasy purification kit (Qiagen, Valencia, CA). The tissue was homogenized in QIAzol Lysis Reagent (cat-79306) with Qiagen TissueLyser II with metal beads in 1 mL of QIAzol Lysis Reagent. To 1 mL of homogenate, 200 µl of chloroform was added and centrifuged at 13,200 rpm for 20 min. The supernatant was collected, and equal volume of 70% ethanol was added. RNeasy purification kit (i.e., RW1 wash buffer and RPE buffer) was used to isolate RNA according to manufacturer’s specification. The purity of RNA was measured by nanodrop-1000 (Thermo Scientific) and 1 µg of RNA was converted to first-strand complementary DNA (cDNA) using high-capacity RNA to cDNA EcoDry™ Premix (Double Primed) reverse transcription kit (cat-639548 Takara Bio USA, Inc). The following primer designs were used for Claudin-5 and Occludin genes while GAPDH was used as housekeeping gene:

**Table:** 

Claudin-5	Forward: TACTCAGCACCAAGGCGAACCAC
	Reverse: GCGGCTT CCCACATCGGTC
Occludin	Forward: AGTACATGGCTGCTGCTGATG
	Reverse: CCCACCATCCTCTTGAT GTGT
GAPDH	Forward: TGCCAGCCTCGTCTCATAG
	Reverse: ACTGTGCCGTTGAACTTGC

Real time-PCR was performed at 95 °C for 10 min followed by 45 cycles of 95 °C for 30 s, 60 °C for 60 s, and 72 °C for 30 s in Applied Biosystems StepOnePlus™ Real-Time PCR System (ABI, Carlsbad, CA). The relative mRNA expression levels were determined by calculating the difference in cycle threshold (Ct) values between the target gene and GAPDH gene (i.e., ΔCt = Ct_target − Ct_GAPDH) for each sample. To assess PCR efficiency and validate the quantification, standard curves were generated using a serial dilution of total cDNA. Relative expressions were calculated by comparing the Ct values of the test samples to the corresponding standard curves and normalizing to GAPDH expression levels.

### Data and statistical analysis

Most behavioral test results collated from ANY-maze 7.3 were validated with manual video analysis for accuracy and consistency. GraphPad Prism version 10.5 (403) was used for statistical analysis of data. Outliers were identified using Grubb’s test. Descriptive statistics were used to summarize the data. Data were analyzed between and/ or within groups using unpaired student’s t-test or 2-way analysis of variance (ANOVA), as appropriate. Post-hoc analyses were conducted using uncorrected Fisher’s least significant difference (LSD) multiple comparisons where necessary. All data are expressed as mean ± standard deviation (SD). Significance was set at P < 0.05.

## Results

### Blood pressure values for SHR and WKY are consistent with strain-specific phenotypes

**Fig. 2:**
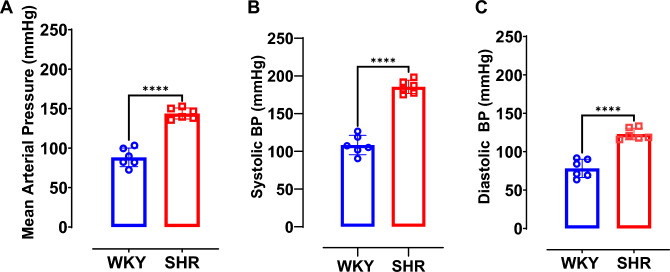
Blood pressure values for SHR and WKY are consistent with strain-specific phenotypes. In vivo hemodynamics measurement was performed via right common carotid artery catheter insertion. (**A**) Mean arterial blood pressure. (**B**) Systolic blood pressure. (**C**) Diastolic blood pressure. Data were collected from male WKY (n = 6) and SHR (n = 6) rats. Values are expressed as mean ± SD. ****P < 0.0001.

When compared to age-matched male WKY rats, SHR show a significantly higher MAP (p = 0.0001; Fig. 2A), systolic blood pressure (SBP; p = 0.0001; Fig. 2B) and diastolic blood pressure (DBP; p = 0.0001; Fig. 2C). The results were obtained from randomly selected cohorts of both SHR and WKY rats. This data set confirms that the SHR used for this study were of hypertensive phenotype and that the WKY rats were normotensive.

### SHR show higher anxiety-like and exploratory behavior during both light and dark phases.

**Fig. 3:**
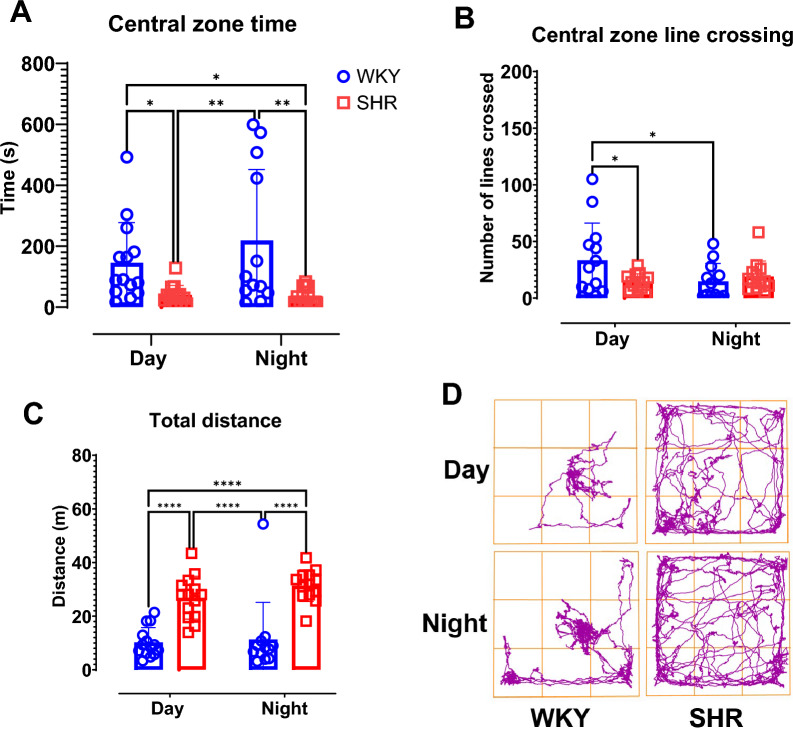
SHR shows higher anxiety-like and exploratory behavior during both light and dark phases. Open field test was performed during the day and during the night for distinct group of rats. (**A**) Time in central zone. (**B**) Central zone line crossing. (**C**) Total distance. (**D**) Representative track plot per group. Data were collected from male WKY (n = 12–14) and SHR (n = 12–14) rats per phase. Values are expressed as mean ± SD. *P < 0.05, **P < 0.01, ****P < 0.0001.

Open field test central zone time shows that WKY rats spend more time in the central zone during light (p = 0.038) and dark (p = 0.001) phases when compared to SHR (Fig. 3A). There was a strain specific difference as shown by 2-way ANOVA (*F* (1, 49) = 15.84, p = 0.0002) for central zone time. The results for time (*F* (1, 49) = 0.943, p = 0.34) and interaction between time and strain (*F* (1, 49) = 1.121, p = 0.3) were not statistically significant. This indicates a strain-specific factor for differences in central zone time during both light and dark phases with SHR spending significantly lower time in the central zone of the open field apparatus (Fig. 3A).

In Fig. 3B, uncorrected Fisher’s least significant difference (LSD) shows significantly higher central zone line crossings in WKY than in SHR during light phase (p = 0.02) and significantly greater line crossings for WKY during light phase when compared to night phase (p = 0.02). Two-way ANOVA shows statistical significance (*F* (1, 48) = 4.59, p = 0.04) in the interaction between strain and time. Moreover, there was no statistical significance for time (*F* (1, 48) = 1.604, p = 0.21) and strain (*F* (1, 48) = 1.83, p = 0.18) per se.

However, SHR covered more distances in the open field apparatus when compared to WKY rats during both light (p = 0.0001) and dark (p = 0.0001) phases (Fig. 3C). Two-way ANOVA reveals a strain-specific factor (*F* (1, 49) = 58.16, p = 0.0001) and there was no statistical significance for time (*F* (1, 49) = 1.348, p = 0.25) or the interaction between time and strain (*F* (1, 49) = 0.494, p = 0.49).

### Recognition memory increased during dark phase for WKY rats

**Fig. 4:**
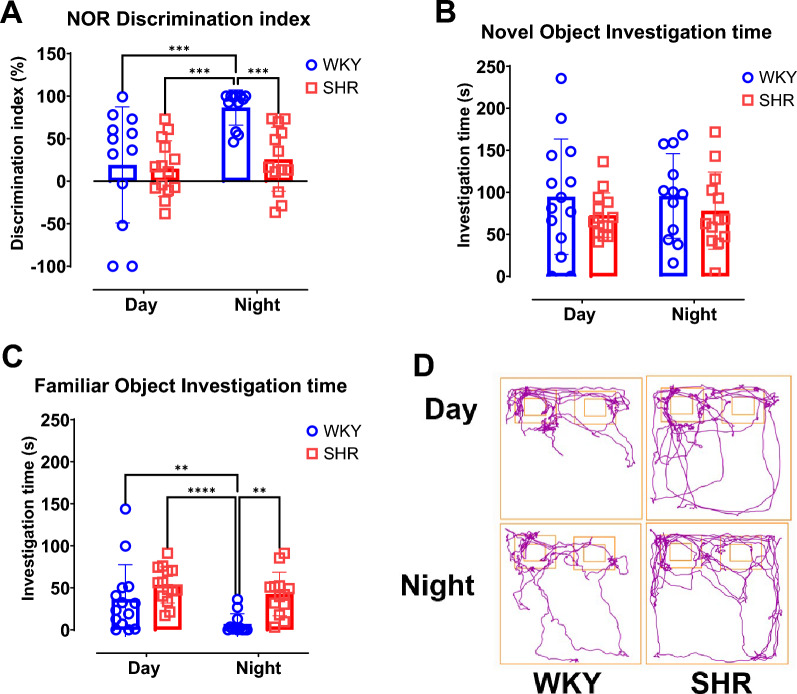
Recognition memory increased during dark phase for WKY rats. Novel object recognition test was performed during the day and during the night for distinct group of rats. (**A**) Novel object discrimination index (**B**) Novel object investigation time. (**C**) Familiar object investigation time D. Representative track plot of Novel object recognition test per group. Data were collected from male WKY (n = 12–14) and SHR (n = 12–14) rats per phase. Values are expressed as mean ± SD. **P < 0.01, ***P < 0.001.

The percentage discrimination index of the NOR test reveals that recognition memory increased in WKY rats during dark phase when compared to data acquired during light phase (p = 0.0004; Fig. 4A). However, recognition memory for SHR does not vary by time of day (p = 0.513). Hence, during the light phase, there is no significant statistical difference between WKY and SHR (p = 0.796) but during the dark phase there was a statistically higher recognition memory for WKY rats than for SHR (p = 0.0009). Further, 2-way ANOVA shows a statistical significance for strain (*F* (1, 47) = 7.325, p = 0.009), time (*F* (1, 47) = 10.54, p = 0.002) and the interaction between time and strain (*F* (1, 47) = 5.486, p = 0.024).

For novel object investigation time (Fig. 4B), there is no statistical difference between WKY and SHR during light (p = 0.25) or dark (p = 0.38) phases. Two-way ANOVA shows no statistical significance for strain (*F* (1, 49) = 2.029, p = 0.16), time (F (1, 49) = 0.0516, p = 0.82) or the interaction between time and strain (F (1, 49) = 0.02589, p = 0.87). However, there is a statistically significant decrease in familiar object investigation time from light phase to dark phase for WKY (p = 0.009; Fig. 4C). Also, there is a statistically significant difference between SHR and WKY for familiar object investigation time during dark phase (p = 0.002). Two-way ANOVA shows statistical significance for strain (*F* (1, 49) = 11.88, p = 0.001) and time (*F* (1, 49) = 7.008, p = 0.011) but not the interaction between time and strain (*F *(1, 49) = 1.506, 0.23).

### Higher spatial memory observed for SHR during both dark and light phases

**Fig. 5:**
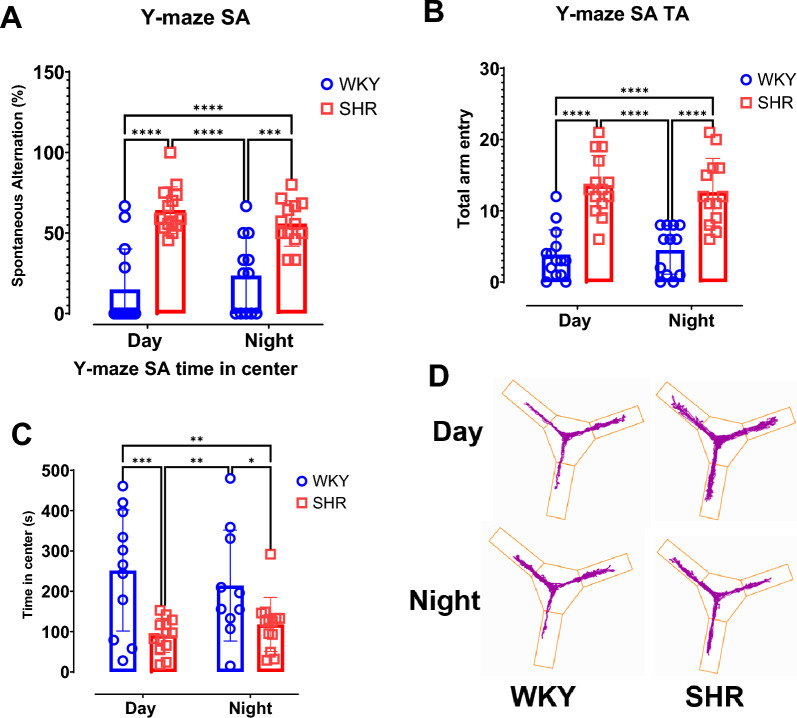
Higher spatial memory observed for SHR during both dark and light phases. Y-maze Spontaneous alternation test was performed during the day and during the night for distinct group of rats. (**A**) Spontaneous alternation. (**B**) Total arm entry. (**C**) Time in center. (**D**) Representative track plot of Y-maze spontaneous alternation test per group. Data were collected from male WKY (n = 12–14) and SHR (n = 12–14) rats per phase. Values are expressed as mean ± SD. **P < 0.01, ***P < 0.001.

Y-maze percentage spontaneous alternation data (Fig. 5A) reveal a significantly higher spatial memory in SHR when compared to WKY during both light (p < 0.0001) and dark (p = 0.0002) phases. Two-way ANOVA demonstrates a strain-specific significance (F (1, 48) = 54.7, p < 0.0001) and no statistical significance for time (F (1, 48) = 0.0004, p = 0.98) nor the interaction between time and strain (*F* (1, 48) = 2.365, p = 0.131). Similarly, SHR exhibit significantly higher total arm entries when compared to WKY for both light (p < 0.0001; Fig. 5B) and dark (p < 0.0001) phases. Two-way ANOVA demonstrates a strain-specific significance (F (1, 49) = 65.78, p < 0.0001) and no statistical significance for time (*F* (1, 49) = 0.020, p = 0.89) nor the interaction between time and strain (*F (1, 49)* = *0.529*, p = 0.47).

Nevertheless, WKY spent significantly more time in Y-maze center zone when compared with SHR during light (p = 0.001) and dark (p = 0.034) phases. Two-way ANOVA demonstrates a strain-specific significance (F (1, 43) = 17.55, p = 0.0001) and no statistical significance for time (F (1, 43) = 0.028, p = 0.87) nor the interaction between time and strain (F (1, 43) = 1.105, p = 0.299).

### SHR shows depression-like behavior during the light phase but a higher immobility episode during dark phase

**Fig. 6:**
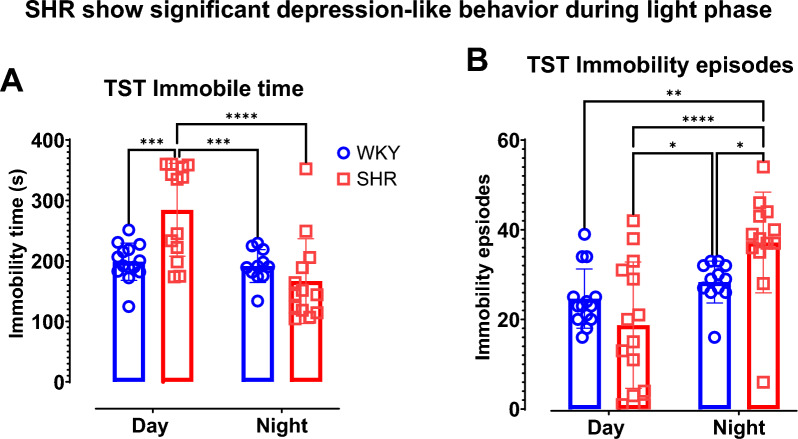
SHR shows depression-like behavior during the light phase but a higher immobility episode during dark phase. Tail suspension test was performed during the day and during the night for distinct group of rats. (**A**) Immobility time. (**B**) Immobility episodes. Data were collected from male WKY (n = 12–14) and SHR (n = 12–14) rats per phase. Values are expressed as mean ± SD. *P < 0.05, ***P < 0.001, ****P < 0.0001.

Immobility time of the tail suspension test was significantly higher in SHR during light phase when compared to WKY (p = 0.0003; Fig. 6A). There was no statistically significant difference in immobility time between SHR and WKY during dark phase (p = 0.289). Two-way ANOVA reveals a significant factor of time (F (1, 47) = 15.57, P = 0.0003) and interaction between time and strain (F (1, 47) = 12.15, p = P = 0.001). However, there is a marginal trend of statistical significance for strain factor (F (1, 47) = 3.705, p = 0.06).

For immobility episodes, there was no significant difference between SHR and WKY during light phase (p = 0.123) but there was a statistically significant difference between SHR and WKY during dark phase (p = 0.0323). Two-way ANOVA shows a significant factor of time (F (1, 49) = 16.16, p = 0.0002) and interaction between time and strain (F (1, 49) = 7.176, p = 0.01). However, there was no statistical significance for strain factor (F (1, 49) = 0.276, p = 0.6).

### No strain difference in BBB permeability index and gene expressions for BBB tight junction proteins

**Fig. 7:**
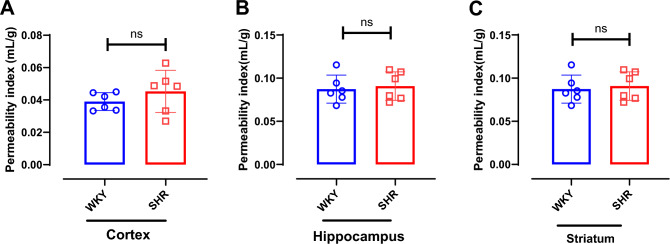
No strain difference in BBB permeability index. Permeability index was measured for (**A**) cortex, (**B**) hippocampus and (**C**) striatum. Data presented as Mean ± SD; n = 6 per strain. ns-non-significant.

BBB function was assessed by PI of FITC-Dextran in the cortex, hippocampus and striatum. There is no statistically significant difference in PI of hippocampus (p = 0.723) and striatum (p = 0.723) between SHR and WKY (Fig. 7). However, though not statistically significant, PI in the cortex of SHR is marginally higher (p = 0.0791) than WKY.

**Fig. 8:**
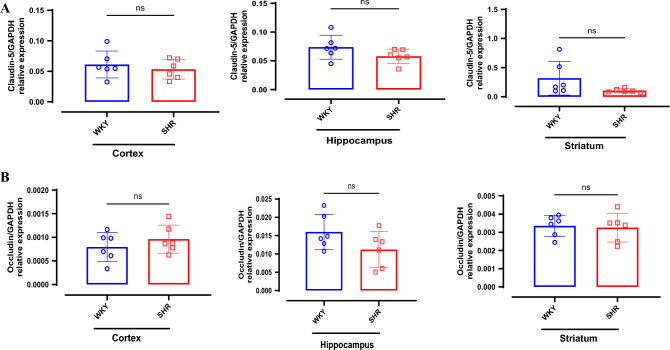
No strain difference in gene expressions for BBB tight junction proteins. RT-QPCR for claudin-5 and Occludin genes was measured in different brain regions. (**A**) Gene expression for Claudin-5 in cortex, hippocampus and striatum. (**B**) Gene expression for Occludin in cortex, hippocampus and striatum. Data presented as Mean ± SD; n = 6 per strain. ns-non-significant.

RT-qPCR analyses revealed no statistically significant differences between SHR and WKY for Claudin-5 in the cortex (p = 0.499, hippocampus (p = 0.144) and striatum (p = 0.1; Fig. 8). Likewise, there was no statistically significant difference between SHR and WKY for Occludin mRNA in the cortex (p = 0.36), hippocampus (p = 0.1) and striatum (p = 0.8111).

### Significantly decreased BBB tight junction proteins but increased FITC-Dextran signal in SHR.

**Fig. 9:**
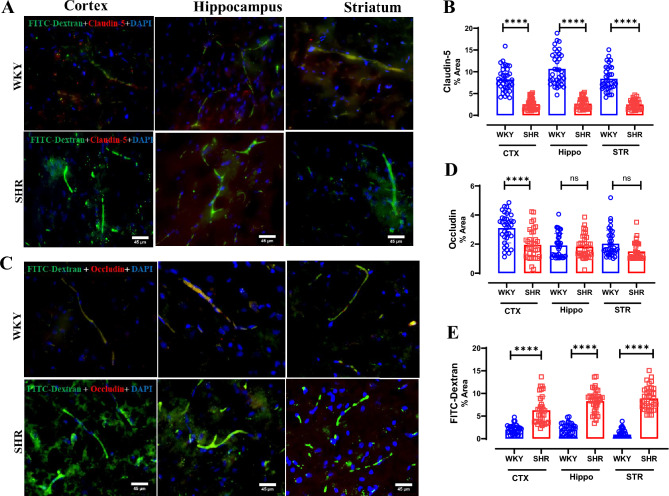
Significantly decreased BBB tight junction proteins but increased FITC-Dextran signal in SHR. (**A**) Representative ×20 magnification micrograph of cortex, hippocampus and striatum staining for Claudin-5 and FITC-Dextran strain in WKY (top row) and SHR (bottom row). (**B**) Quantification for claudin-5 in % area of fluorescent intensity. (**C**) Representative ×20 magnification micrograph of cortex, hippocampus and striatum staining for Occludin and FITC-Dextran strain in WKY (top row) and SHR (bottom row). (**D**) Quantification for Occludin in % area of fluorescent intensity. (**E**) Quantification for FITC-Dextran in % area of fluorescent intensity. Micrographs were analyzed from six sections per rat and n = 6 per strain. Scale bar = 45 µm. Data presented as Mean ± SD. ****P < 0.0001.

Immunoreactivity for Claudin-5 is significantly decreased in cortex (p < 0.0001), hippocampus (p < 0.0001) and striatum (p < 0.0001) in SHR when compared to WKY rats (Fig. 9). Similarly, in the cortex of SHR immunoreactivity for Occludin is significantly decreased (p < 0.0001) when compared to WKY (Fig. 9C). However, there is no statistically significant difference between SHR and WKY rats in the immunoreactivity for Occludin in hippocampus (p = 0.64) and striatum (p = 0.008); Fig. 9C). Micrographs of the cortex, hippocampus and striatum as well as their respective quantification of FITC-Dextran reveal significant (p < 0.0001) BBB extravasation in SHR when compared to WKY rats (Fig. 8A and 9A).

## Discussion

The present report reveals diurnal differences in cognitive and mood-associated behavior for adult male SHR and WKY rats. Firstly, compared to light phase, WKY rats exhibit significantly higher recognition memory during the dark phase than SHR which had preserved recognition memory during both phases. This implies that the NOR test is best conducted for male WKY and SHR during their active phase rather than during their inactive phase. Secondly, SHR showed significantly elevated Y-maze working memory when compared to WKY rats during both light and dark phases. Thirdly, SHR demonstrate significant depression-like behavior during light phase when compared to dark phase and with WKY rats at both phases. During the dark phase, even with significantly increased immobility episodes, SHR have comparable total immobility time as WKY rats. Fourthly, at both phases, we confirm significantly higher locomotor activity for SHR than WKY rats. Lastly, histology of the cortex, hippocampus and striatum of SHR demonstrates compromised BBB integrity, when compared with WKY rats.

The purview of this study is to determine whether there is a day-night difference in neurobehavioral test outcome between 6 months old male SHR and WKY rats. Consistent with established day time test reports^13,34^, the total distance from our open field test and Y-maze total arm entry data confirm that SHR have significantly higher locomotor activity at both light and dark phases than WKY rats. Increased locomotor activity in SHR has been associated with poor spatial memory test performance^35–37^; and suggested to be the cause for distraction which results in working memory impairment. However, in our hands, even with increased locomotor activity, when compared to male WKY rats, SHR had significantly higher working memory on the Y-maze during both light and dark phases. This finding is consistent with other types of spatial working memory such as a hole board test^36^ and 12-arm radial maze test^38^. The latter^38^ further highlighted that SHR excelled at learning to vary responses but failed at repeat responses. However, since the Y-maze spontaneous alternation test performance is based on avoiding repeated arm entry, the actual driving factor for better working memory performance in our male SHR and WKY rats is elusive. Previous studies^39–41^ suggest that hypertension alone does not impair cognition, but advanced hypertension with aging in SHR is necessary for learning impairment in younger rats. These studies^39–41^ demonstrate early aging-related cognitive impairment in SHR since 3–4 months old SHR performed cognitively better than 12 or 16–17 months old SHR using radial arm maze learning tasks or autoshaping learning and activity tasks. Conversely, in a 28-day deoxycorticosterone acetate (DOCA)-salt model of hypertension, mice showed significant cognitive dysfunction using NOR and Barnes maze tests indicating that salt sensitive hypertension can induce early memory deficits^42^. Also, a report on water-based radial maze test for working memory showed that 3 months old SHR exhibited memory deficits when compared to WKY rats^37^. Water-based memory tests provide intrinsic motivation for rodents to learn how to escape due to their aversion for water, thus providing reliable test measures. However, ‘dry’ working memory tests such as Y-maze spontaneous alternation are simpler and time-effective as it bypasses several days of training the animals. The use of either type of working memory tests would depend on the rodent age and specific research questions which the experimenter seeks to investigate. Here, we preferred 6 months of age as it represents a timepoint of advanced chronic hypertension in SHR after blood pressure stabilization at 4–5 months of age. Also, this age reflects valid adulthood for both SHR and WKY rats before age-related cognitive impairment sets in.

In tandem with published reports for higher impulsiveness in SHR compared to WKY rats^7,43^, we show that at both time-of-day WKY rats spent more time in the Y-maze center area than SHR. Increased time in the Y-maze center area can be associated with non-impulsivity and high attentive behavior which is indicative of better decision-making**.** We concede that this observation can be attributed to the lack of motivation for WKY rats to explore the maze compared to SHR. Due to their increased locomotor activity, SHR would typically spend less time in the center.

We show that time-of-day does not change recognition memory for SHR with the NOR test, suggesting that recognition memory is preserved in SHR and is not subject to change by time of day. However, recognition memory is significantly higher in WKY rats during the dark phase when compared to light phase. Our day-time result is in contrast with a report^44^ which showed that seven and half months old SHR had significantly decreased cognitive function on the NOR test when compared to WKY. While there is no prior report for NOR comparison in the dark phase between SHR and WKY rats, in agreement with our day-time data, a recent day-time study demonstrated that there was no difference between SHR and WKY rats in the first 5 min of discrimination test^13^. However, this test was performed 60 min after the familiarization phase unlike ours which was started in half the time. The authors^13^ showed that allowing the animals an additional 5 min, but not 10 min, in the apparatus revealed a significant decrease in cognition for SHR as compared with WKY rats. Whether or not additional time in the discrimination phase would reveal between group differences for recognition requires further confirmation. During the dark phase, WKY rats spent significantly less time exploring the familiar object when compared to light phase investigation time, showing time of day bias against familiar object exploration. The significantly decreased time spent by WKY rats during the dark phase exploring the familiar object drives our observations for a true time of day difference, rather than strain-specific differences in recognition memory. Together, although WKY rats are less motivated to explore the open field, their preference to not explore the familiar object during the day indicates an actual recognition of the novel object.

Since recognition memory in SHR is relatively preserved in addition to a very high working memory, we contend that cognition in SHR at six months of age is related to BBB leakage which is widely reported in this strain^44–47^ and other hypertensive models^48,49^. Our histological observation of high FITC-Dextran expression as well as significant decrease in Claudin-5 and Occludin for SHR when compared with WKY, suggest BBB extravasation and compromised integrity. This is consistent with other reports on BBB leakage in the hypothalamus of SHR^44–47^. Even so, we infer that the genes coding for Claudin-5 and Occludin are being transcribed at the same rate, given the similar gene expression levels in SHR and WKY. Comparable mRNA expressions for both strains do not automatically guarantee similar amounts of tissue protein due to potential strain differences in translation efficiency, protein degradation rates and post-translational modifications. Similarly, the PI which underscores functional BBB permeability did not differ between strains across brain regions. We concede that this may be due to the lack of transcardial perfusion which would have cleared intravascular FITC-Dextran thereby resulting in similar residual tissue concentration. It is not well understood whether the basic biology of BBB protein translation is impaired in cognition-related brain areas of SHR and whether functional BBB permeability differences between strains warrant future clarification. In essence, regardless of compromised BBB integrity in brain areas relevant to cognition, memory function in six months old SHR is stable.

WKY rats are described as a model of depression-like behavior^11,50,51^. At variance with our day-time data, SHR exhibited significantly decreased immobility in the forced swim test (FST) when compared to WKY rats^11^. While there is no prior report on TST for measuring depression-like behavior in SHR or WKY rats, our data reveal a significant day-time depression-like behavior in SHR when compared with WKY rats. In our hands, this implies that the optimal time for TST following experimental intervention is during the dark phase when WKY and SHR would otherwise have comparable values. Both FST and TST are established measures of despair or depression-like behavior in rodents, hence the varying outcome between the present observation and another report^11^ may be age-related. The main difference between our study and the FST report is the age of the rats used: our study employed 6-month-old rats, whereas the FST report^11^ utilized rats aged 6–7 weeks.

The order in which a battery of behavioral tests proceed could significantly impact cognitive and affective behavior in rodents. Here, we conducted our behavioral test protocols beginning with cognitive function assessments and concluded with TST in accordance with best practice of least to most complex/ stressful^52–55^. Y-maze is less complex than NOR which must be preceded by OFT while TST is more stressful for rodents. In addition, to decrease carry over effects, we allowed at least one day of recovery time between Y-maze, NOR and TST. Hence, we contend that a different order of behavioral test performed in this context would yield more interpretable findings.

There are several limitations associated with this study. Firstly, we did not include age-matched female rats for data collection. Even though, sex is a biological variable, the majority of studies which reported cognitive and mood-associated data using SHR and WKY rats used only males. Hence, future studies ought to investigate whether there is a sex-specific difference in the diurnal outcomes of neurobehavioral tests. Secondly, infrared light during the dark phase would have been ideal to maintain the animals’ natural nocturnal behavior in the dark since rodents cannot see infrared light^56^. For consistency in testing room illumination between light and dark phases, we used dim visible light at 22 Lux. However, visible light can potentially distort rodents’ nocturnal response^56^ and may constrain the interpretation of our findings. Future endeavors should clarify whether visible light differs for test outcomes when compared to infrared light. Thirdly, we did not include Wistar rats as it was outside the purview of the present study aimed at assessing time-of-day behavioral test outcomes between SHR and the genetically similar but normotensive control WKY rats. Wistar rat groups may have provided a balanced context for strengthening the interpretation of our behavioral data. Lastly, our study focused only on 6 months old SHR and WKY rats without consideration for advanced age. It is established that both SHR and WKY show features of cognitive impairments with advancing age; more so in SHR, however, whether time of day when cognitive tests are performed makes a difference is for future studies to investigate.

## Conclusion

To the best of our knowledge, this is the first report showing that there is a significant day-night outcome difference for recognition memory and depression-like behavior in young male SHR and WKY rats. This suggests that following experimental manipulations, NOR and TST are best performed during the dark phase. Moreover, we demonstrate that at 6 months of age, male SHR show significantly compromised BBB integrity particularly in the cortex and striatum. Since working memory is preserved in SHR, it implies that with chronic hypertension at 6 months of age, impaired BBB integrity did not drive cognitive or depression-like behavioral differences between SHR and WKY.

## Data Availability

The data which supports the findings of this study are available from the corresponding author on request.
